# Therapeutic Applicability of Anti-Inflammatory and Proresolving Polyunsaturated Fatty Acid-Derived Lipid Mediators

**DOI:** 10.1100/tsw.2010.57

**Published:** 2010-04-13

**Authors:** Gerard L. Bannenberg

**Affiliations:** Centro Nacional de Biotecnología/CSIC, Department of Plant Molecular Genetics, Campus de la Universidad Autónoma, Madrid, Spain

**Keywords:** anti-inflammatory, apoptosis, aspirin, inflammation, leukocyte, lipid mediator, protectins, resolution, resolvins

## Abstract

The enzymatic oxygenation of polyunsaturated fatty acids by lipoxygenases and cyclo-oxygenases is a resourceful mode of formation of specific autacoids that regulate the extent and pace of the inflammatory response. Arachidonate-derived eicosanoids, such as lipoxin A_4_, prostaglandin (PG)D_2_, PGF_2_α, PGE_2_, and PGD_2_-derived cyclopentenones exert specific roles in counter-regulating inflammation and turning on resolution. Recently recognized classes of autacoids derived from long-chain ω-3 polyunsaturated fatty acids, the E- and D-series resolvins, protectin D1, and maresin 1, act as specialized mediators to dampen inflammation actively, afford tissue protection, stimulate host defense, and activate resolution. It is held that counter-regulatory lipid mediators and the specific molecular pathways activated by such endogenous agonists may be suitable for pharmacological use in the treatment of inflammatory disease. The anti-inflammatory drug aspirin is a striking example of a drug that is able to act in such a manner, namely through triggering the formation of 15-epi-lipoxin A_4_ and aspirin-triggered resolvins. Different aspects of the therapeutic applicability of lipid mediators have been addressed here, and indicate that the development of innovative pharmacotherapy based on anti-inflammatory and proresolution lipid mediators presents novel prospects for the treatment of inflammatory disease.

## THE INFLAMMATORY RESPONSE

### Inflammation

The healthy state is the default condition of living organisms, provided that the right physical, nutritional, and emotional circumstances are present. As a result of dealing with unequal distribution and limiting amounts of essential resources, the continuous competition and interaction between organisms is a constant in life. In animals and humans, innate and adaptive immune responses have evolved to allow the body to deal at a tissue- and organism-wide level with intra- and interspecies interactions on a background of environmental changes. When the physical integrity of an organism is violated due to infection or wounding, a physiological response is called into action, namely the inflammatory response[1,2]. This response effectively deals with removing the inciting infection or tissue damage, is self-limiting, and allows the body to recover the healthy state. Counter-regulatory pathways built into the inflammatory response allow a careful balance between mounting a sufficiently strong response to clear infections, yet limiting damage to endogenous tissue. Without endogenous control over the inflammatory response, the natural state of the biotic world would be sickness.

Inflammation is characterized by five cardinal attributes: *calor* (heat that results from increased tissue perfusion and an altered body temperature set point), *rubor* (redness that results from increased perfusion of superficial tissue), *tumor* (swelling of the inflamed tissue as a result of plasma exudation), *dolor* (inflammatory pain and increased sensitivity to sensory nerve simulation), and *functio laesa* (diminished tissue function). These characteristics reflect events that occur at the local tissue level as well as centrally mediated actions that allow the body to act as an integrated system, to adjust its physiology in order to deal with incurred damage and infection[3,4]. A marked infection or trauma also triggers an acute phase reaction, which is sensed at the organism level as sickness and is characterized by fever, anorexia, and decreased physical activity and interest[5].

Research over the past century has gradually increased our knowledge of the cellular and molecular events that take place during the mounting of the inflammatory response. The principal objective of the inflammatory response is delivery of blood-borne phagocytes to the site of damage/infection, with the aim to clear the inciting stimulus and, when completed, initiate a return to tissue homeostasis. All the necessary information for triggering an appropriate inflammatory response is likely to be provided by the type of interaction, which is recognized by innate and adaptive immune repertoires of sentinel systems that alert to specific types of injury or interspecies interactions[6,7,8].

The directed migration of blood-borne leukocytes to the inflammatory locus, a transient increase in vascular permeability that facilitates plasma exudation, and phagocytosis of microbes and dying cells are central events during the proinflammatory phase of the inflammatory response[9,10]. During the time course of inflammation, specific cells are recruited and distinct molecular mechanisms activated in a temporal and tissue-specific fashion. In a classical view, polymorphonuclear leukocytes are the first cells recruited from blood to the perturbed tissue, followed by infiltration of monocytes that differentiate into macrophages. Tissue-specific differences exist in the magnitude of the inflammatory response, the specific subset of inflammatory leukocytes involved, and the mediators that are employed in regulating the different phases of the inflammatory response[11,12,13,14]. The inflammatory response resembles the execution of a molecular and cellular program that passes through checkpoints that function to assess the nature of the insult, the progress of leukocyte accumulation and microbial clearance, and to initiate resolution and tissue repair[11,15,16,17].

## Resolution

Successful clearance of infecting microbes and damaged tissue is normally followed by disappearance of the inflammatory exudate, a process called resolution. Resolution of inflammation has in recent years been recognized to be a highly regulated physiological process in itself, and can be considered an integral part of the inflammatory response[11,18]. After their specific functions have been completed, neutrophils die by apoptosis and are removed by phagocytosis, or disappear from the inflammatory exudate by migration via the lymphatics or towards an epithelial surface[2,10,15,19,20]. Phagocytosis of apoptotic neutrophils by macrophages constitutes a key mechanism whereby an inflamed tissue switches from a proinflammatory state to one that begins to resolve, as it leads to a reduction of macrophage proinflammatory mediator formation and to the release of anti-inflammatory and resolution-promoting lipid mediators and cytokines[21,22,23,24]. Such nonphlogistic phagocytosis can be viewed as an example of a heterocellular interaction between neutrophils and macrophages, providing information on the temporal progress of the inflammatory response, which is then used towards reducing the extent of inflammation and promoting resolution.

Although researchers considered resolution of inflammation an adequate term for naming the end of inflammation, it is only recently that resolution has started to be considered an actively regulated part of the inflammatory response, involving the activation of specific molecules and cells that signal the end of inflammation and turn it off[16,25,26]. This is a radical change in point of view, with important implications in understanding the physiology behind the inflammatory response, and it permits revisiting the mechanisms of inflammatory pathophysiology. As diverse sources of tissue perturbation, malfunction, and infection likely require activation of different sets of inflammatory mediators to direct adequate cellular responses for eliminating the inciting stimulus, it is foreseeable that the resolution of inflammation also employs specific mechanisms to return to homeostasis. The directed activation of resolution allows placing control over specific molecular pathways that need to be activated in order to guide an inflamed tissue back to its original functional state.

Specific endogenously generated autacoids are now known to regulate the counter-regulation and resolution of inflammation[27,28,29,30,31]. Cellular events that are activated during resolution include the active reduction of further inflammatory cell recruitment through sequestration of proinflammatory chemokines via clearance receptors and down-regulation of adhesion molecules, the promotion of lymphatic egress of leukocytes, the transepithelial migration of leukocytes to mucosal surfaces from where the cells can be cleared, the stimulation of removal of apoptotic cells by phagocytosis, and the restoration of damaged tissue structures[20,21,27,32,33]. Specific members of the eicosanoids, arachidonic acid (AA)–derived lipid mediators, are known to function as endogenous counter-regulatory and proresolution mediators, or stimulate anti-inflammatory actions upon administration[25,34]. Recently, enzymatically oxygenated lipid mediators derived from ω-3 polyunsaturated fatty acids (PUFAs), such as eicosapentaenoic acid (EPA) and docosahexaenoic acid (DHA), have been identified to play central roles in the endogenous regulation of the inflammatory response[27]. These mediators, named resolvins, protectins, and maresins, constitute a recently identified group of autacoids with potent anti-inflammatory, tissue-protective, and resolution-stimulating functions.

## INFLAMMATION AND DISEASE

Persistent or recurring components of inflammation are now recognized to characterize many human disorders, collectively termed inflammatory disease. Unrelenting exposure to noxious stimuli, deficiencies in specific nutrients, and hereditary or acquired defects in the inflammatory response can cause or predispose to a range of diseases that display specific features of inflammation. Particular aspects of inflammation and/or damage incurred from activated neutrophils or macrophages are present in common diseases such as atherosclerosis, stroke, asthma, rheumatoid arthritis, Alzheimer's disease, retinopathies, periodontitis, and Crohn's disease[35,36,37,38,39,40,41,42,43,44,45].

Tissue damage is widely considered to be an inevitable result of the inflammatory response, as neutrophils are believed to lack absolute specificity in their function to kill microbes and consequently “spill” cytotoxic mediators, such as reactive oxygen and nitrogen species, proteases, and hypochlorite formed by released myeloperoxidase; neutrophil microbicides kill not only pathogens, but also damage surrounding tissue[34,45,46,47,48,49]. In a normal inflammatory response, locally damaged tissue will normally heal efficiently, yet a temporary loss in tissue function can ensue from the activated neutrophils during inflammation. In chronic inflammatory diseases, tissue damage incurred during the persisting presence of activated neutrophils may underlie the pathology of the disease, often due to remodeling of tissue architecture. Disproportionate and unimpaired acute inflammatory responses may also lead to marked tissue damage and failure of multiple organs[50]. Other chronic inflammatory disorders lack involvement of an overt inflammatory response and can be better characterized by a low-grade, chronic activation of tissue macrophages due to tissue stress or malfunction[51].

Given the burden of acute and chronic inflammatory diseases, the advancement of medicine has historically focused on achieving pharmacological intervention with the objective to reduce the proinflammatory phase of the inflammatory response[52]. Not only in chronic inflammatory diseases, but also in excessive and life-threatening acute inflammation, both a reduction of inflammation and the activation of resolution constitute a desirable outcome, for example, in acute lung injury[53]. An appealing hypothesis that is currently being assessed for its validity entails that derangement of counter-regulatory mechanisms to limit leukocyte infiltration or their activation, or failure of specific autacoids to initiate the resolution of inflammation, constitutes a cause of inflammatory disease[16,54]. The present review therefore aims to provide an overview of our current knowledge, and provides examples, of the use of lipid mediators with anti-inflammatory and proresolution activities to achieve a reduction in inflammatory disease.

## ANTI-INFLAMMATORY AND PRORESOLUTION LIPID MEDIATORS

In order to limit the extent of inflammation, and bring a specific tissue or the body back to the healthy state, the existence of specific sensory mechanisms exist that gauge the progress of the inflammatory response, and that relay information to activate counter-regulatory mechanisms or signal the end of inflammation. To this purpose, the body employs a substantial number of lipid mediators, which are derived from both AA and from the ω-3 PUFAs EPA and DHA. These substances act as autacoids and are endowed with anti-inflammatory and proresolution properties mediated via activation of specific receptor proteins. The actions of these mediators are characterized by regulation at multiple levels of the organism's organization. The best-studied anti-inflammatory/proresolution lipid mediator, lipoxin $A_4$ ($LXA_4$), for example, not only modifies cellular activity of leukocytes via binding of a specific G-protein coupled receptor, but also activates gene transcription[55,56,57], directly regulates intracellular enzyme activity[58], and acts at higher physiological levels to modulate neural transmission and vascular tone[59,60,61,62,63]. It is likely that these actions integrate to modulate physiology in order to achieve a new state of tissue homeostasis.

Recent studies have indicated the existence of specialized proresolution lipid mediators (SPM) that act as endogenously generated lipid mediators to activate the resolution of inflammation[64]. SPMs include ω-3 PUFA–derived resolvins, protectins, and maresins. In addition to SPMs, a number of AA-derived prostaglandins are increasingly recognized also to exert tissue-specific anti-inflammatory and proresolution actions, in addition to their more established roles as proinflammatory lipid mediators. The following section will provide a general overview of the formation and actions of the thus-far-identified PUFA-derived anti-inflammatory/proresolution lipid mediators.

### Arachidonic Acid–Derived Anti-Inflammatory Lipid Mediators

#### *Lipoxin $A_4$*

During interactions of distinct cell types, such as neutrophils and endothelial cells or neutrophils and platelets, the fatty acid AA can undergo a double oxygenation by the sequential action of two different lipoxygenases[65]. This process, termed transcellular biosynthesis, is a fundamental way whereby information on the proximity or tight contact between different cell types is transduced into the enzymatic formation of specific lipid mediators with potent bioactivity. In the case of AA, one of the double oxygenation products has been identified as 5*S*,6*R*,15*S*-trihydroxy-(7*E*,9*E*,11*Z*,13*E*)-tetraenoic acid, or $LXA_4$ (Fig. 1)[66,67]. In humans, transcellular biosynthesis of $LXA_4$ has been demonstrated after percutaneous transluminal coronary angioplasty, and its formation has been measured in many organs[68,69,70]. $LXA_4$ activates a number of molecular events that counteract the further recruitment of neutrophils to an inflamed tissue. This is achieved through down-regulation of adhesion molecules on endothelial cells and leukocytes, as well as by reduced migration towards proinflammatory chemotactic

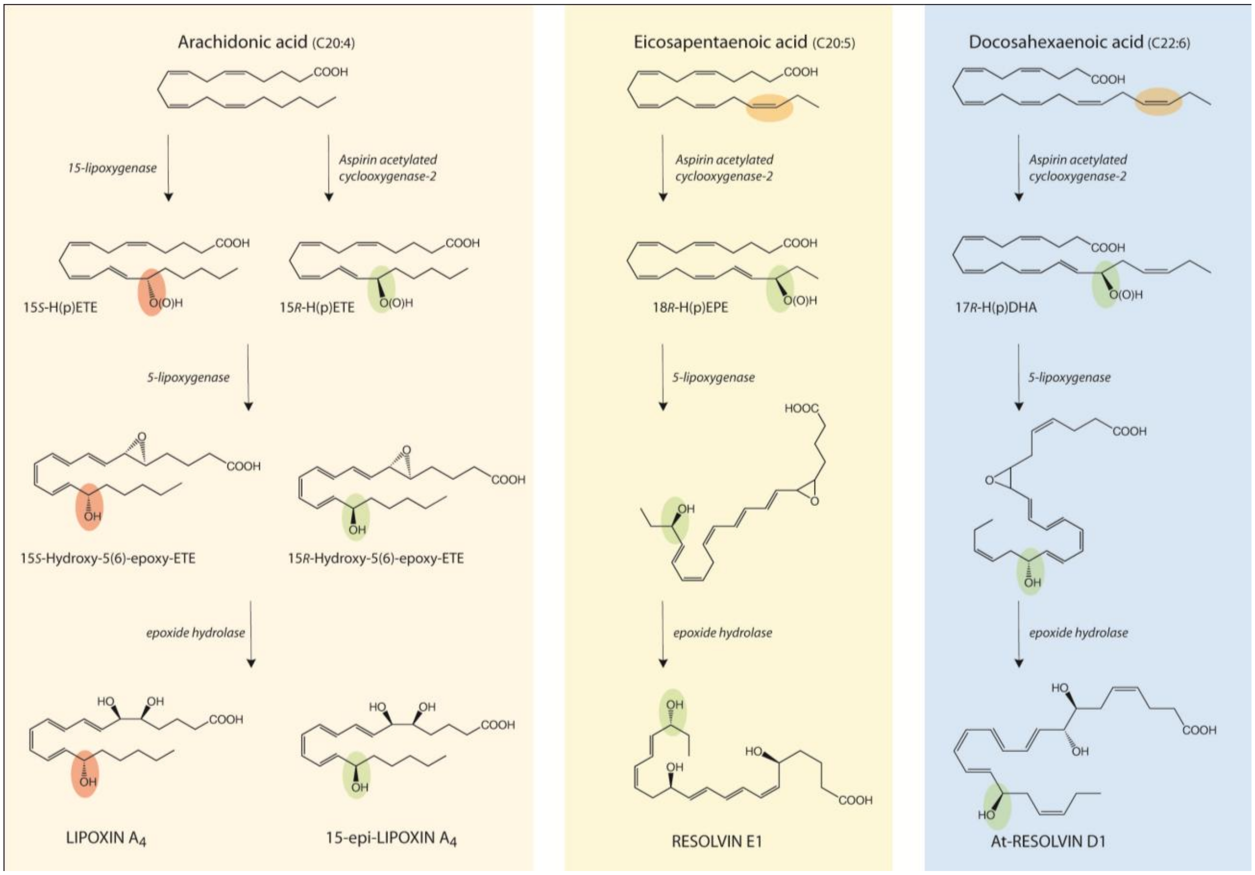

**FIGURE 1.** Epimeric oxygenation of PUFAs in the biosynthesis of anti-inflammatory and proresolving lipid mediators. After acetylation by aspirin, COX-2 directs the formation of 15*R*-hydroperoxy-eicosatetraenoic acid (15*R*-HpETE), 18*R*-hydroperoxy-eicosapentaenoic acid (18*R*-HpEPE), and 17*R*-hydroperoxydocosahexaenoic acid (17*R*-HpDHA) via an epimeric oxygenation (indicated in green). The stereospecificity of molecular oxygen incorporation catalyzed by fatty acid oxygenases occurs typically in the S-configuration (red), as indicated for $LXA_4$ biosynthesis. The primary oxygenation products 15*R*-HpETE, 18*R*-HpEPE, and 17*R*-HpDHA are subsequently oxygenated by 5-lipoxygenase (which is abundant in neutrophils) to form the anti-inflammatory and proresolving lipid mediators 15-epi-$LXA_4$, resolvin E1 (RvE1), and aspirin-triggered resolvin D1 (At-RvD1), respectively. Omega-3 fatty acids, such as EPA and DHA, are characterized by an ω-3 double bond (yellow) located three carbons away from the methyl end of the chemical structure, whereas the final double bond in the ω-6 PUFA AA is located six carbons from the ω-end of the molecule. The biosynthesis of 15-epi-$LXA_4$ is similar to that of $LXA_4$, with the important difference that COX-2 participates in the biosynthesis of 15-epi-$LXA_4$. Note that there is a second biosynthetic pathway for $LXA_4$ formation (not shown), which involves the 5-lipoxygenase–mediated formation of leukotriene $A_4$ followed by the action of a 12-lipoxygenase to form $LXA_4$[315].

gradients[71,72,73]. $LXA_4$ also reduces the activation state of neutrophils, measurable by a decreased respiratory burst and degranulation[74,75,76], and reduces proinflammatory cytokine release in different models of inflammation[74,77]. $LXA_4$ stimulates the progress of the inflammatory response towards resolution by activating monocyte migration to the inflamed tissue, overriding prosurvival signals to favor neutrophil apoptosis, and stimulating nonphlogistic phagocytosis by monocytes/macrophages of dying neutrophils that have completed their actions as phagocytes of tissue-infecting microbes[21,78,79]. The anti-inflammatory actions of $LXA_4$ have been demonstrated in a number of tissues, including the airways, kidney, liver, gastrointestinal tract, and brain[80,81]. Such activating actions are receptor-mediated; $LXA_4$ is an agonist of the formyl peptide receptor (FPR)2/ALX, a G-protein coupled receptor ($K_d \approx 0.7$ nM)[82].

Lipoxin $B_4$ (5S,14*R*,15S-trihydroxy-6*E*,8*Z*,10*E*,12*E*-eicosatetraenoic acid) is a positional isomer of $LXA_4$ that is also formed by transcellular lipoxygenase biosynthetic pathways[83]. $LXB_4$ has been shown to share many of the anti-inflammatory actions activated by $LXA_4$ *in vivo*, but via activation of distinct signal transduction pathways through a yet-unidentified G-protein coupled receptor[78,84,85].

## *Prostaglandin $E_2$*

$PGE_2$ is an eicosanoid generated during inflammation and contributes to the early vasodilatation and capillary permeability that characterize the onset of inflammation (Fig. 2)[86]. $PGE_2$ sensitizes sensory nerve endings and thereby also contributes to inflammatory pain[87,88]. Given these actions, its synthesis during the mounting of inflammation, and the fact that substantial levels of $PGE_2$ can also be observed in chronic inflammation, it is not surprising that this prostaglandin has been historically viewed, for the most part, as a proinflammatory lipid mediator[89,90,91,92,93]. In consonance, a reduction in $PGE_2$ formation is logically observed by nonsteroidal anti-inflammatory drugs (NSAIDs) that inhibit cyclo-oxygenase. A substantial number of studies have also indicated, however, that $PGE_2$ plays distinct roles in down-regulating immune-inflammatory responses, promotes tissue repair, and can activate resolution[91,94,95, 96,97,98,99,100,101].

The tissue-protective and anti-inflammatory roles of $PGE_2$ in the lung have been recognized for quite some time[102,103]. $PGE_2$, acting via activation of the EP4 receptor, has also been shown to contribute to maintaining a noninflammatory milieu in the colon[104]. During infection of rats with the helminth *Angiostrongylus costaricensus*, $PGE_2$ stimulates resolution of allergic edema and eosinophilic inflammation[105]. $PGE_2$ can be useful to reduce experimental adjuvant-stimulated arthritis[106]. High levels of $PGE_2$ are formed during inflammation, which peak at the onset of resolution in the mouse air pouch model[107,108]. $PGE_2$ has been implicated in so-called class switching of lipid mediator biosynthesis; by activating the transcription of 15-lipoxygenase in exudate neutrophils during tumor necrosis factor-α (TNF-α)–induced inflammation, $PGE_2$ helps in establishing a complete biosynthetic pathway for $LXA_4$ in neutrophils (which also express 5-lipoxygenase). The generation of $LXA_4$ upon its turn can activate the resolution of inflammation[108].

## *Prostaglandin $D_2$*

$PGD_2$ (Fig. 2) is also known to exert inflammation-limiting and proresolution actions. $PGD_2$ can reduce neutrophil infiltration in the early phases of experimentally induced colitis in rats[109]. Local administration of fibroblasts expressing hematopoietic PGD synthase, one of the $PGD_2$ biosynthetic enzymes, induces a reduction of neutrophilic inflammation in the murine air pouch model[110]. Specific functions for $PGD_2$ in inflammation resolution have been demonstrated by a number of studies. The level of $PGD_2$ is markedly increased in inflammatory exudates just prior to and during resolution in zymosan-stimulated murine peritonitis[92,111]. The expression of hematopoietic PGD synthase is increased during resolution of inflammation of cardiac tissue after endotoxin treatment of mice[112]. In addition, inhibition of $PGD_2$ formation, through inhibition of cyclo-oxygenase 2 (COX-2), impairs the resolution of carrageenin-stimulated pleurisy in mice[113].

Not much is known with respect to the importance of activation of the known $PGD_2$ receptors, DP1 and CRTH2/DP2, for their involvement in resolution. $PGD_2$ is an anti-inflammatory lipid mediator formed during the early phase of an acute inflammatory response (in zymosan-stimulated peritonitis)[114]. DP1 receptor stimulation is important for this anti-inflammatory action and also activates the disappearance of a resident lymphocyte population during inflammation[114]. As these lymphocytes play an important regulatory role in the formation of cytokines that direct the proinflammatory infiltration of neutrophils, their disappearance is likely to be of importance for permitting subsequent resolution to take place. Subsequent lymphocyte repopulation of a resolved site of inflammation is important for being capable of responding to subsequent infections with a normal inflammatory response[114].

The study of the role of $PGD_2$ in inflammation has developed into a significant topic of research since nonenzymatic breakdown products of $PGD_2$ were recognized to exert potent anti-inflammatory actions[115,116,117,118]. These products are prostaglandins with a cyclopentenone structure and include

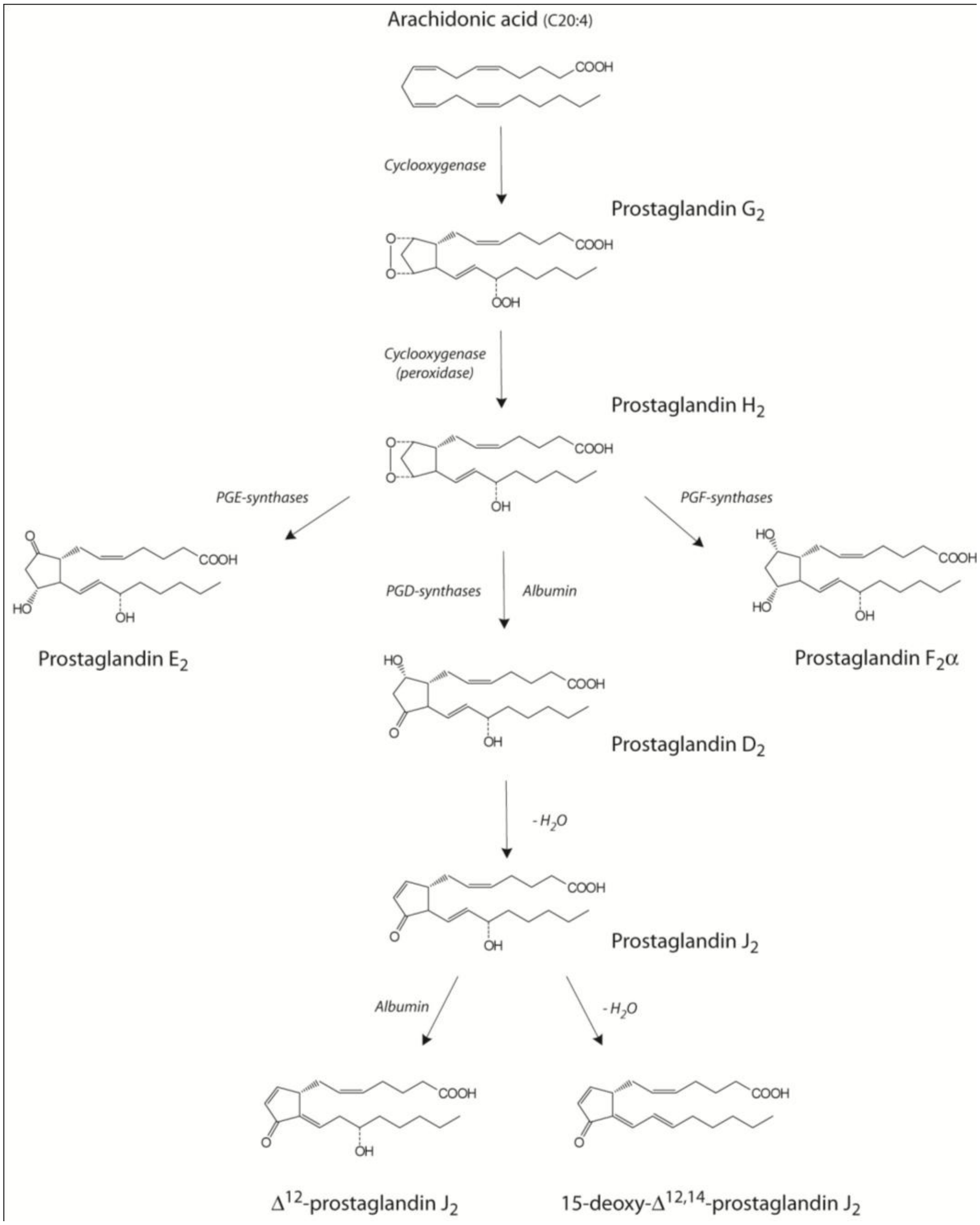

**FIGURE 2.** Formation of prostaglandins that are currently known to possess anti-inflammatory and proresolving activity. Several AA (C20:4)-derived prostaglandins are known to exert specific anti-inflammatory and proresolution roles. Note that EPA and DHA can also produce prostaglandins, which are analogous to those derived from AA, but these are generally not considered to constitute specific endogenous receptor ligands.

$PGJ_2$, $\Delta^{12}$-$PGJ_2$, and 15-deoxy-$\Delta^{12,14}$-$PGJ_2$ (Fig. 2). The chemical reactivity of cyclopentenone prostaglandins with sulfhydryl groups defines a different mechanism of action than anti-inflammatory lipid mediators that act via interactions with G-protein coupled receptors. A number of redox-sensitive transcription factors are known to be regulated after covalent modification by cyclopentenone prostaglandins, including PPARγ, Nrf2/Keap1, NF-κB, AP-1, H-Ras, and p53[119,120,121], subsequently activating the transcription of genes and activating a heat shock response, which confer tissue protection during the inflammatory response[117,122,123]. $PGD_2$-derived compounds can regulate the intensity of delayed-type hypersensitivity responses and act as endogenous braking signals for lymphocytes to stimulate resolution[124]. Mice deficient in hematopoietic PGD synthase display defects in the resolution of T-cell–dominated inflammation in a model of delayed-type hypersensitivity[124]. Administration of 15-deoxy-$\Delta^{12,14}$-$PGJ_2$ stimulates resolution in these mice, indicating that $PGD_2$-derived cyclopentenone products are capable of terminating inflammation. $\Delta^{12}$-$PGJ_2$ and 15-deoxy-$\Delta^{12,14}$-$PGJ_2$ also stimulate neutrophil and macrophage apoptosis, and their formation is triggered during phagocytosis of apoptotic neutrophils by macrophages[125,126,127]. Cyclopentenone-containing lipid products can also be formed through dehydration reactions from $PGE_2$[128], as well as by the nonenzymatic oxidation of PUFAs such as AA[129].

At elevated concentrations, α,β-unsaturated aldehyde-containing lipids can directly contribute to tissue damage[130,131]. At low concentrations, the presence of such reactive lipid mediators is sensed and believed to be employed as signaling molecules, which act either directly, via activation of sensor proteins that activate gene transcription to enhance tissue protection, or indirectly, where reaction with low-molecular-weight thiols, such as glutathione, may activate an inflammatory response[132]. The latter may serve as a signal to recruit phagocytes for the removal of oxidized tissue in a wound. A useful mind-set may be to accept that organisms do employ both receptor-mediated actions and actions via reactive alkylation of regulatory sensors to inform cells on the state of tissue perturbation. Covalent modification of regulatory proteins by reactive cyclopentenones indicates that not all agonists for resolution obligatorily act through reversible high-affinity interactions with cell-surface G-protein coupled receptors. The physiological basis that underlies these distinct types of signaling mechanisms may be based on the value in being able to sense both enzymatically formed mediators and chemically reactive lipid mediators that relay distinct types of information about the insult that triggers tissue perturbation and inflammation. Whereas eicosanoid formation can be activated by cellular recognition of a pathogen-associated molecular fragment, and transcellular biosynthesis of lipoxins and resolvins informs on cellular interactions during inflammation, the formation of reactive lipids is often the result of free radical generation, hydrogen abstraction, and subsequent peroxidation of lipids. This can be a consequence of, for example, tissue decompartmentalization (loss of cellular membrane organization), redox-cycling agents, ionizing radiation, or activation of a respiratory burst[133,134].

The reason why $PGD_2$-derived dehydration products are employed as reactive enones to promote resolution is less clear. Possibly this pathway specifically senses the high levels of extravasated plasma proteins, especially albumin, present in an inflammatory exudate. Albumin has been shown to be capable of supporting the transformation of $PGD_2$ to this type of lipid mediator[135,136]. Also, the formation of $PGD_2$ from $PGH_2$ can be catalyzed by albumin and plasma proteins[137], indicating that the entire biosynthetic, but nonenzymatic, pathway from $PGH_2$ towards sulfhydryl-reactive cyclopentenones can be catalyzed by plasma proteins (Fig. 2).

## Prostaglandin $I_2$

Several studies have indicated that $PGI_2$ (prostacyclin) may also act as an endogenous regulator of inflammation. Through activation of PPAR-α, $PGI_2$ can reduce NF-κB–mediated TNF-α formation after ischemia/reperfusion (I/R) injury of the kidney[138]. This is relevant as PPAR-α activation has been demonstrated to contribute to the resolution of inflammation in kidney I/R[139]. $PGI_2$ has also been indicate to activate PPAR-γ through stimulation of IP-receptor signaling[140]. Activation of the IP receptor with a receptor agonist can facilitate the expression of genes induced by the anti-inflammatory glucocorticoid dexamethasone in human bronchial epithelial cells[141]. In hepatic I/R, $PGI_2$ formation is augmented and has been suggested to reduce neutrophil activation[142]. Direct actions on leukocytes have been demonstrated as well. Prostacyclin analogs inhibit the release of TNF-α from monocytic cells[143] and can reduce neutrophil activation[144].

## *Prostaglandin $F_2\alpha$*

The possible anti-inflammatory and proresolution roles of $PGF_2\alpha$ (Fig. 2) have been insufficiently studied with respect to the resolution of inflammation. $PGF_2\alpha$ is implicated to function as an endogenous agonist for resolution of pleural inflammation in mice, as the selective FP receptor agonist fluprostenol can reverse an exacerbation of murine pleural inflammation during the resolution phase after inhibition of COX-2 with a highly selective COX-2 inhibitor[145].

## *15-Epi-$LXA_4$*

The NSAID aspirin acetylates the active site of cyclo-oxygenase enzymes, thereby inhibiting the biosynthesis of $PGH_2$, the central endoperoxide precursor for eicosanoid biosynthesis (Fig. 2). In the case of the COX-2 isoenzyme, the modification of the active site changes the binding of AA in such a way that it allows a single oxygenation reaction, but with altered stereospecificity of oxygen incorporation, leading to formation of 15*R*-hydroperoxy-eicosatetraenoic acid (15*R*-HETE; Fig. 1)[146]. In contrast, acetylation of COX-1 by aspirin does not allow such a change in catalytic activity and is believed to inhibit all possible ways of oxygenation of AA.

Subsequent transformation of 15*R*-HETE by 5-lipoxygenase–containing human neutrophils leads to the generation of 15-epi-$LXA_4$[147] (Fig. 1). This aspirin-triggered $LXA_4$ (ATL) isomer, 15-epi-$LXA_4$, also acts via activation of FPR2/ALX to counteract the migration and activation of neutrophils, down-regulates proinflammatory cytokine release, and promotes monocyte/macrophage functions, such as migration and the nonphlogistic phagocytosis of apoptotic cells[82,148]. Also, aspirin triggers 15-epi-$LXA_4$ formation in humans[149,150]. Recently, low doses of orally administered aspirin have been shown to reduce leukocyte infiltration significantly in skin blisters, stimulate 15-epi-$LXA_4$ formation, and increase FPR2/ALX expression[150]. Due to the epimeric configuration of the 15-hydroxyl group, 15-epi-$LXA_4$ is more resistant than $LXA_4$ to enzymatic dehydrogenation of the 15-hydroxyl group by the enzyme 15-hydroxyprostaglandin dehydrogenase (PGDH) to form biologically inactive 15-oxo-$LXA_4$[151]. Resistance to this route of metabolic inactivation makes 15-epi-$LXA_4$ a significantly more potent anti-inflammatory mediator *in vivo* than $LXA_4$[152].

The awareness that a small change in the chemical structure of $LXA_4$ affords increased anti-inflammatory activity *in vivo* has led to two developments. First, it has stimulated the rational design of stable $LXA_4$ analogs, which can be administered to experimental animals and retain or display enhanced, anti-inflammatory activity. A pharmacological assessment of distinct lipoxin analogs has shown that resistance to metabolic inactivation by PGDH, cytochrome $P_{450}$-mediated ω-oxidation[153], and β-oxidation can be achieved while maintaining biological activity[154,155]. One group of stable pharmacological agonists are the ATL analogs that retain the biological activity of $LXA_4$[156,157]. A frequently employed stable ATL analog, 16-(*p*-fluorophenoxy)-15-epi-$LXA_4$-methyl ester (ATLa2), with potent anti-inflammatory actions *in vivo* is shown in Fig. 3 (compound **1**). Stable and active analogs of aspirin-triggered $LXB_4$, such as 15-epi-$LXB_4$-methyl ester, have also been designed[158].

Second, the formation of 15-epi-$LXA_4$ called for the question whether aspirin-triggered 15-epi-lipoxin formation reflects the existence of an endogenous mode of 15-epi-$LXA_4$ formation. It has recently been indicated that nitric oxide (NO) may constitute this endogenous equivalent and likely acts through modification of the COX-2 enzyme to direct the epimeric oxygenation of AA, analogous to the way aspirin-mediated COX-2 acetylation triggers this pathway[159,160]. These findings indicate that modulation of endogenous lipid mediator stability may be employed actively by the body to enhance their counter-regulatory potency, likely when the formation of NO is activated in response to inflammatory stimuli.

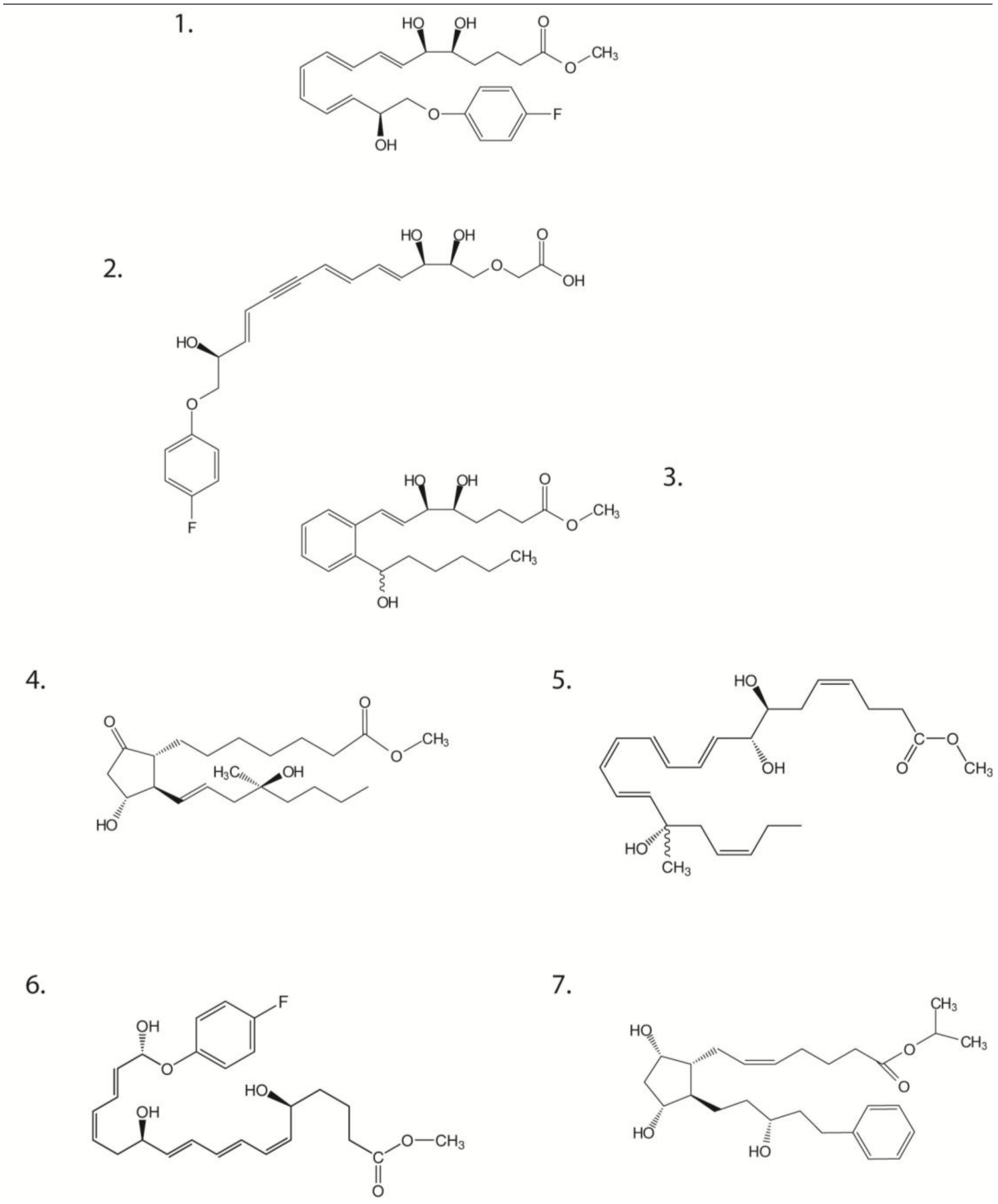

**FIGURE 3.** Chemical structures of selected lipid mediator stable analogs. **1.** 16-(*p*-Fluorophenoxy)-15-epi-$LXA_4$-methyl ester (ATLa2); **2.** ZK994, a trienyne/3-oxa analog of 16-(*p*-fluorophenoxy)-15-epi-$LXA_4$; **3.** a benzo-$LXA_4$ analog; **4.** misoprostol; **5.** 17-(*R*/*S*)-methyl-RvD1 carboxymethyl ester; **6.** 19-(*p*-fluorophenoxy)-RvE1 carboxymethyl ester; **7.** latanoprost.

## Omega-3 Fatty Acid–Derived Anti-Inflammatory Lipid Mediators

Recent studies have demonstrated that the ω-3 PUFAs EPA and DHA (Fig. 1) are also substrates for enzymatic oxygenation reactions to form lipid mediators with potent anti-inflammatory and proresolution actions. This knowledge was not obvious as little as 15 years ago because the cyclo-oxygenase enzymes that were known to oxygenate AA to form $PGH_2$, the central fatty acid hydroperoxide precursor for the biosynthesis of the prostaglandins and thromboxane $A_2$, had been found to oxygenate EPA or DHA at relatively low rates and the products do not activate the known receptors for eicosanoids with sufficient affinity to be recognized as endogenously relevant mediators[161,162,163,164,165].

Omega-3 fatty acids are endogenous polyunsaturated long-chain fatty acids that are essential for human health[166,167,168,169,170,171]. Phospholipids containing acylated EPA and DHA constitute a significant percentage of the fatty acid composition in specific locations in the body, e.g., the central nervous system, the retina, and sperm cells[172,173]. Omega-3 fatty acids make an important contribution to structural and functional roles of specific subcellular membrane compartments[174,175]. It is now well documented that dietary ω-3 fatty acids impart protective actions in the cardiovascular and nervous systems, and can counteract a range of inflammatory diseases[27,166,167,168,169,170,171].

A substantial body of evidence exists that indicates that the composition of the average occidental diet is currently profoundly skewed towards a relatively high proportion of omega-6 (ω-6) fatty acids, such as linoleic acid and AA, compared with ω-3 fatty acids, such as α-linolenic acid, EPA, and DHA. Our current diet is markedly different from the natural diet that humans consumed for tens of thousands of years, which likely consisted of a relatively easily accessible and ample range of food types present at coastal, fluvial, and lake areas in Southern and Eastern Africa[176]. In particular, it provided a diet rich in fish and shellfish full of ω-3 PUFAs, which has been proposed to have permitted the distinctive cognitive development that is characteristic for human beings[177]. Nowadays, the inability of the majority of humans who have adapted to a "western" lifestyle to consume this habitual diet may be causing a chronic deprivation of sufficient ω-3 fatty acid intake. Only very recently have dietary recommendations started to aim for a more balanced ratio of dietary ω-3 fatty acid to ω-6 fatty acid consumption[178,179].

A re-evaluation of the results of a large epidemiological study that precisely aimed to assess the impact of enhanced ω-3 fatty acid intake on mortality and recurring heart attacks in patients who had experienced a prior myocardial infarction strongly suggested that the enrolled patients might not only have experienced the salutatory effects of increased ω-3 fatty acid intake, but were also taking aspirin as a cardiovascular protection agent[180]. As a result of this observation, Serhan and colleagues addressed the possibility that ω-3 fatty acids EPA and DHA may also constitute substrates for aspirin-acetylated COX-2 and form novel oxygenated lipid mediators, analogous to the formation of 15-epi-$LXA_4$ from AA. This line of research has revealed a large new family of EPA- and DHA-derived lipid mediators, named resolvins, protectins, and maresins, with potent counter-regulatory properties in the inflammatory response. These lipid mediators act as autacoids, and are formed via specific cellular and transcellular enzymatic pathways[181,182]. The biosynthesis, as well as their cellular and *in vivo* actions, of these novel ω-3–derived lipid mediators have recently been reviewed[26,183].

## E-Series Resolvins

Endothelial cells expressing COX-2 (after activation by TNF-α) and treated with aspirin were able to convert EPA to 18*R*-hydroxy-5*Z*,8*Z*,11*Z*,14*Z*,16*E*-eicosapentaenoic acid (18*R*-HEPE), in a manner similar to the epimeric oxygenation of AA. 18*R*-HEPE could be further oxygenated by 5-lipoxygenase–expressing human neutrophils to form 5*S*,12*R*,18*R*-trihydroxy-6*Z*,8*E*,10*E*,14*Z*,16*E*-eicosapentaenoic acid, a lipid mediator named resolvin E1 (RvE1; Fig. 1)[184]. The name resolvin is derived from the formation of the compound during the 'resol'ution phase of inflammation as a product that is formed during cell-cell 'in'teractions. Human plasma levels of RvE1 are detectable up to 0.4 μg/l after the dietary intake of EPA and DHA together with aspirin[185]. Employing experimental models of self-resolving inflammation (murine air pouch and zymosan-stimulated peritonitis), RvE1 was demonstrated to reduce neutrophilic inflammation potently and activate the resolution of inflammation[111,186]. RvE1 reduces neutrophil-endothelial cell interactions and transmigration, stimulates nonphlogistic phagocytosis by macrophages, and reduces the release of proinflammatory cytokines[33,111,184,185,187,188,189,190,191]. RvE1 also potently inhibits inflammatory angiogenesis[192,193].

The high *in vivo* potency and absolute requirement for the specific stereochemistry and double-bond geometry of RvE1 pointed to highly selective recognition by an endogenous receptor. A G-protein coupled receptor for RvE1 ($K_d$ ~11 nM) was identified in a screen for orphan receptors as CMKLR1 (chemokine-like receptor-1, previously also named ChemR23), a receptor that also binds the peptide chemerin[185,194]. The functional importance of the RvE1-CMKLR1 interaction has been demonstrated thus far in murine dendritic cells (down-regulation of IL-12 formation and migration)[185] and in oral epithelial cells where activation of CMKLR1 by RvE1 stimulates transepithelial neutrophil migration[20]. A second receptor interaction has been identified that mediates other actions of RvE1, namely partial agonism of RvE1 at the leukotriene $B_4$ ($LTB_4$) receptor BLT1 ($K_d$ ~ 45 nM). Displacement of $LTB_4$ constitutes an important mechanism whereby RvE1 dampens the proinflammatory actions of $LTB_4$[184,195]. In summary, RvE1 constitutes the first identified ω-3 fatty acid–derived lipid mediator with receptor-mediated anti-inflammatory/proresolving actions, and acts on two receptors, CMKLR1 and BLT1[185].

The potent anti-inflammatory actions of RvE1 have been demonstrated in a rabbit model of *Porphyromonas gingivalis*–induced periodontitis; the topical application of RvE1 to inflamed gingival tissue resulted in the restoration of lost bone and connective tissue and markedly reduced inflammation[196]. RvE1 exerts anti-inflammatory and tissue-protective actions in I/R-induced kidney injury[197,198]. In white adipose tissue, RvE1 can down-regulate obesity-induced inflammation, which corrects a loss of insulin sensitivity in experimental obesity and abrogates hepatic steatosis[199]. In a murine model of allergic asthma, RvE1 promotes the resolution of allergic airway inflammation through the down-regulation of IL-17, a cytokine that can maintain allergen-induced airway inflammation[200]. RvE1 also down-regulated IL-6 and IL-23, cytokines that are important in stimulating Th17-type responses. Through stimulating IFNγ formation, RvE1 also contributed to resolution of airway inflammation by facilitating apoptosis[200].

The fatty acid hydroperoxide formed by the action of 5-lipoxygenase, 5*S*-hydroperoxy-18*R*-HEPE, can also undergo reduction to a dihydroxy fatty acid named RvE2 (5*S*,18*R*-dihydroxyeicosapentaenoic acid)[201]. RvE2 constitutes a second member of the EPA-derived (E-series) resolvins and likely regulates inflammatory targets that differ from those activated by RvE1[201]. The mechanism whereby 5-lipoxygenase directs RvE2 formation vs. the formation of RvE1 has not been demonstrated, but RvE2 formation may be favored under hypoxic conditions.

## D-Series Resolvins

Once the first evidence had been obtained that EPA could serve as a substrate for the formation of the anti-inflammatory and proresolution lipid mediator RvE1, subsequent experiments were performed to assess the possibility that DHA also could be transformed by aspirin-acetylated COX-2 and 5-lipoxygenase into novel oxygenated products. This was indeed the case, leading to the description of the aspirin-triggered D-series resolvins (AT-RvD)[188,202]. Acetylated COX-2 was shown to catalyze the incorporation of molecular oxygen at carbon 17 of DHA, forming 17*R*-hydroxydocosa-(4*Z*,7*Z*,10*Z*,13*Z*,15*E*,19*Z*)-hexaenoic acid after reduction of the 17*R*-hydroperoxy intermediate. Given the three remaining pentadienyl moieties present in the 17*R*-hydroperoxy intermediate, there are a number of potential lipoxygenase products that could be formed via subsequent oxygenation steps. 5-Lipoxygenase has been shown to catalyze oxygenation at one of two carbons, namely at C4 and C7, leading to the formation of four structurally distinct lipid mediators termed At-RvD1–4. Oxygenation at carbon 7 and subsequent formation and hydrolysis of an intermediate epoxide gives rise to At-RvD1 (shown in Fig. 1) and At-RvD2, whereas oxygenation at carbon 4 leads to formation of At-RvD3 and At-RvD4[188,202]. These DHA-derived products display potent anti-inflammatory actions, such as reduced human neutrophil migration and the inhibition of neutrophilic infiltration in murine models of inflammation.

The AT-RvDs thus represent endogenous epimeric forms that are triggered by exposure to aspirin, analogous to the formation of 15-epi-$LXA_4$ and RvE1 (Fig. 1). The subsequent discovery that D-series resolvins are formed endogenously in human whole blood incubated with DHA without need for acetylation of COX-2 by aspirin was significant because it implied a different route of biosynthesis of DHA-derived resolvins (Fig. 4)[203]. Importantly, these D-series resolvins, RvD1–4, possess a C17-hydroxy group with S stereospecificity, and their biosynthesis requires lipoxygenase-mediated oxygenation prior to 5-lipoxygenase action. Studies with murine 12/15-lipoxygenase have indicated that a rate-limiting factor for this biosynthetic pathway is the expression of the 15-lipoxygenase activity that forms 17*S*-hydroperoxydocosa-(4*Z*,7*Z*,10*Z*,13*Z*,15*E*,19*Z*)-hexaenoic acid intermediate[204].

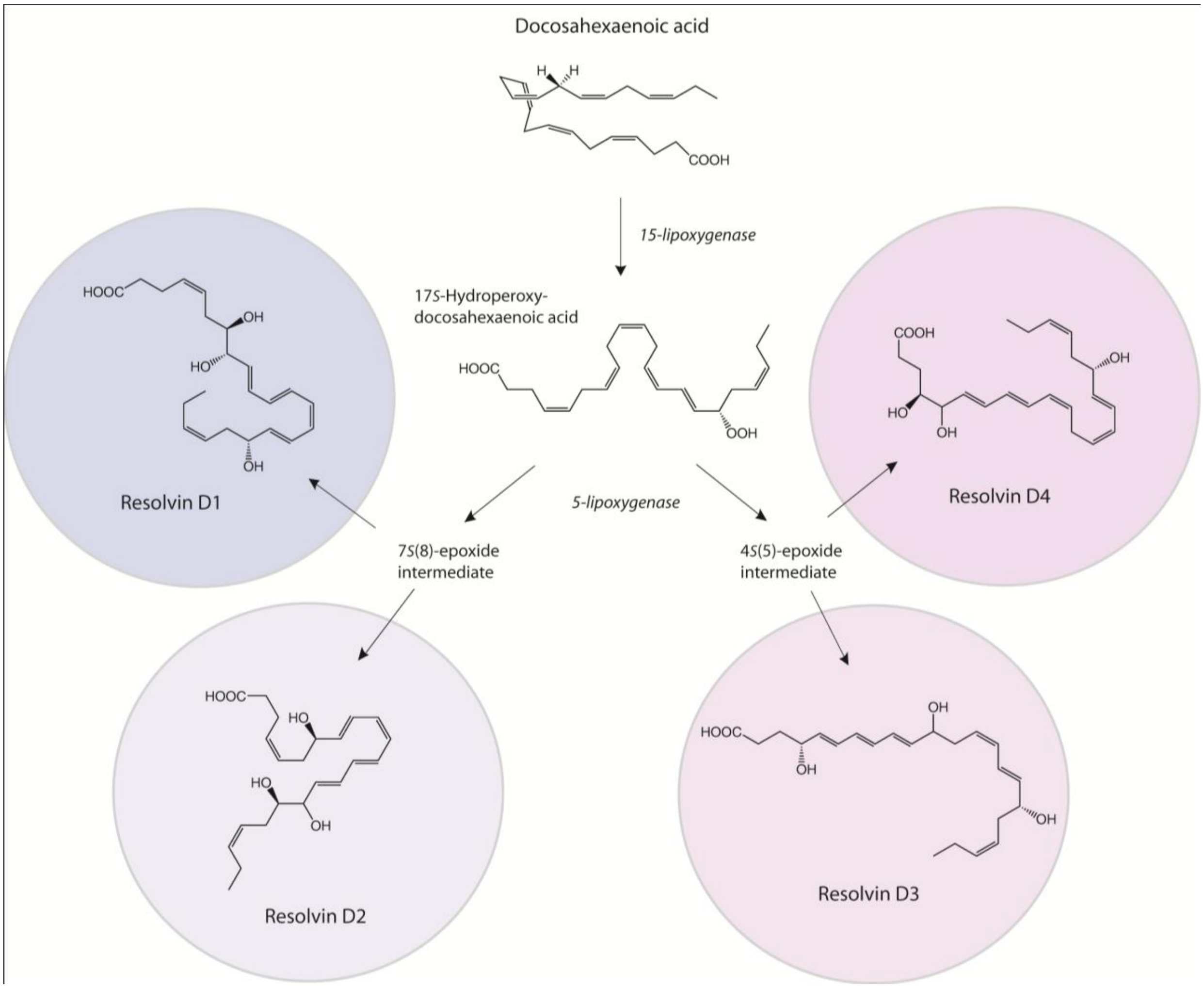

**FIGURE 4.** The biosynthesis of D-series resolvins. Aspirin is not required for the biosynthesis of the DHA (C22:6)-derived (D-series) resolvins D1, D2, D3, and D4 (RvD1–D4). The trihydroxylated D-series resolvins are formed via the intermediate 17S-hydroperoxy-DHA (17*S*-hydroperoxydocosa-(4*Z*,7Z,10*Z*,13*Z*,15*E*,19*Z*)-hexaenoic acid), which is generated via the action of 15-lipoxygenase on DHA, followed by a second 5-lipoxygenase–catalyzed oxygenation that introduces molecular oxygen at C4 or C7 to form epoxide intermediates, which are hydrolyzed and rearranged to the respective resolvins.

RvD1 exerts potent anti-inflammatory and proresolving actions in a number of cell types and models of inflammation[202,203]. RvD1 has been demonstrated in experimental periodontitis in the rabbit and I/R-induced kidney injury in mice[196,197,198]. RvD1 has recently been shown to reduce potently the inflammatory response and neutrophil oxidative burst that are generated in response to the 4-hydroxynonenal-glutathione adduct, a product that reflects the glutathione-dependent detoxification of a reactive lipid aldehyde generated during lipid peroxidation. This tissue-protective response activated by RvD1 possibly represents a mechanism to counteract oxidative tissue injury caused by neutrophil-mediated employment of oxygen and nitrogen radicals in the biocidal removal of infecting microbes[132].

Recent studies have shown that RvD2 has potent regulatory actions on neutrophil trafficking, and stimulates resolution and host defense[205]. The RvD2-stimulated inhibition of neutrophil-endothelial cell interactions and neutrophil trafficking were shown to be mediated, at least in part, by NO. Of importance, this study revealed a novel function of RvD2; namely, the potent activation of microbial phagocytosis by monocytes/macrophages. In an experimental murine sepsis model, RvD2 down-regulated proinflammatory cytokine release and also inhibited release of IL-10, a cytokine that is detrimental in this excessive inflammatory response. The enhanced *in vivo* clearance of bacteria demonstrated that RvD2 promotes host defense and allowed the survival of animals that would normally succumb to the excessive inflammatory response during sepsis. Specific receptor proteins that selectively recognize any of the D-series resolvins remain to be identified.

## Protectin D1 and Maresin 1

In addition to resolvins, DHA can be transformed to additional novel lipid mediators named protectin D1 (PD1) and maresin 1 (MaR1) with potent anti-inflammatory and tissue-protective actions (Fig. 5). The first pathway involves the 17*S*-hydroperoxydocosa-(4*Z*,7*Z*,10*Z*,13*Z*,15*E*,19*Z*)-hexaenoic acid product required for D-series resolvin biosynthesis, and requires enzymatic hydrolysis of an intermediate epoxide, forming 10*R*,17*S*-dihydroxydocosa-(4*Z*,7*Z*,11*E*,13*E*,15*Z*,19*Z*)-hexaenoic acid[206]. This DHA-derived lipid was named protectin D1 because it affords potent tissue-protective actions in brain and retinal cells (PD1 is named neuroprotectin D1 [NPD1] when it is generated by neural cells)[206,207,208].

PD1 exhibits potent anti-inflammatory and tissue-protective activity, and can activate the resolution of inflammation[111,186,203,206]. By down-regulating cell surface adhesion molecules and reducing the release of chemoattractive and neutrophil-activating cytokines, PD1 can potently counter-regulate the transendothelial migration of blood leukocytes to a site of inflammation[33,111,191]. Furthermore, PD1 has been demonstrated to reduce experimental periodontitis in the rabbit and I/R-induced kidney injury[196,197,198], and has been shown to reduce inflammatory angiogenesis and choroidal neovascularization after laser-induced injury of the retina[209,210,211]. In the retina, retinal pigment epithelial cells synthesize PD1 to activate gene expression of cytoprotective genes, which likely contributes to promoting a noninflammatory environment[208,212]. The expression of 12/15-lipoxygenase in macrophages has been shown to aid in protecting mice from atherosclerosis through the formation of $LXA_4$, RvD1, and PD1[204].

In the second pathway, MaR1 (7*S*,14*S*-dihydroxydocosa-(4*Z*,8,10,12,16*Z*,19*Z*)-hexaenoic acid) is formed via the action of 12-lipoxygenase, which oxygenates DHA to form 14*S*-hydroperoxydocosa-(4*Z*,7*Z*,10*Z*,12*E*,16*Z*,19*Z*)-hexaenoic acid intermediate (Fig. 5)[213]. The formation of both the 14*S*-hydroperoxy product and MaR1 has been identified in mouse macrophages during phagocytosis. MaR1 also potently reduces neutrophil migration and stimulates macrophage phagocytosis. Through direct reduction or a second lipoxygenation, additional products can be generated from 14S-hydroperoxydocosahexaenoic acid, thus forming the basis for an entire new family of lipid mediators, termed maresins[213].

In contrast to the double enzymatic oxygenation for resolvin biosynthesis, both PD1 and MaR1 biosynthesis involves a single lipoxygenation step. As transcellular biosynthesis is not required for protectin and maresin biosynthesis, this may indicate that these DHA-derived lipid mediators do not report on transient cellular interactions, but probably integrate extracellular signals received by a single cell type. Recently, a high-affinity binding site of PD1 in a retinal pigment epithelial cell line ($K_d$ = 31.3 pmol/mg cell protein) and in human neutrophils ($K_d \approx$ 25 nM) has been measured[214]. Although the identity of the receptor has not yet been established, no competition with other anti-inflammatory mediators $LXA_4$ or RvE1 was found, indicating that this binding site may constitute a new receptor for PD1.

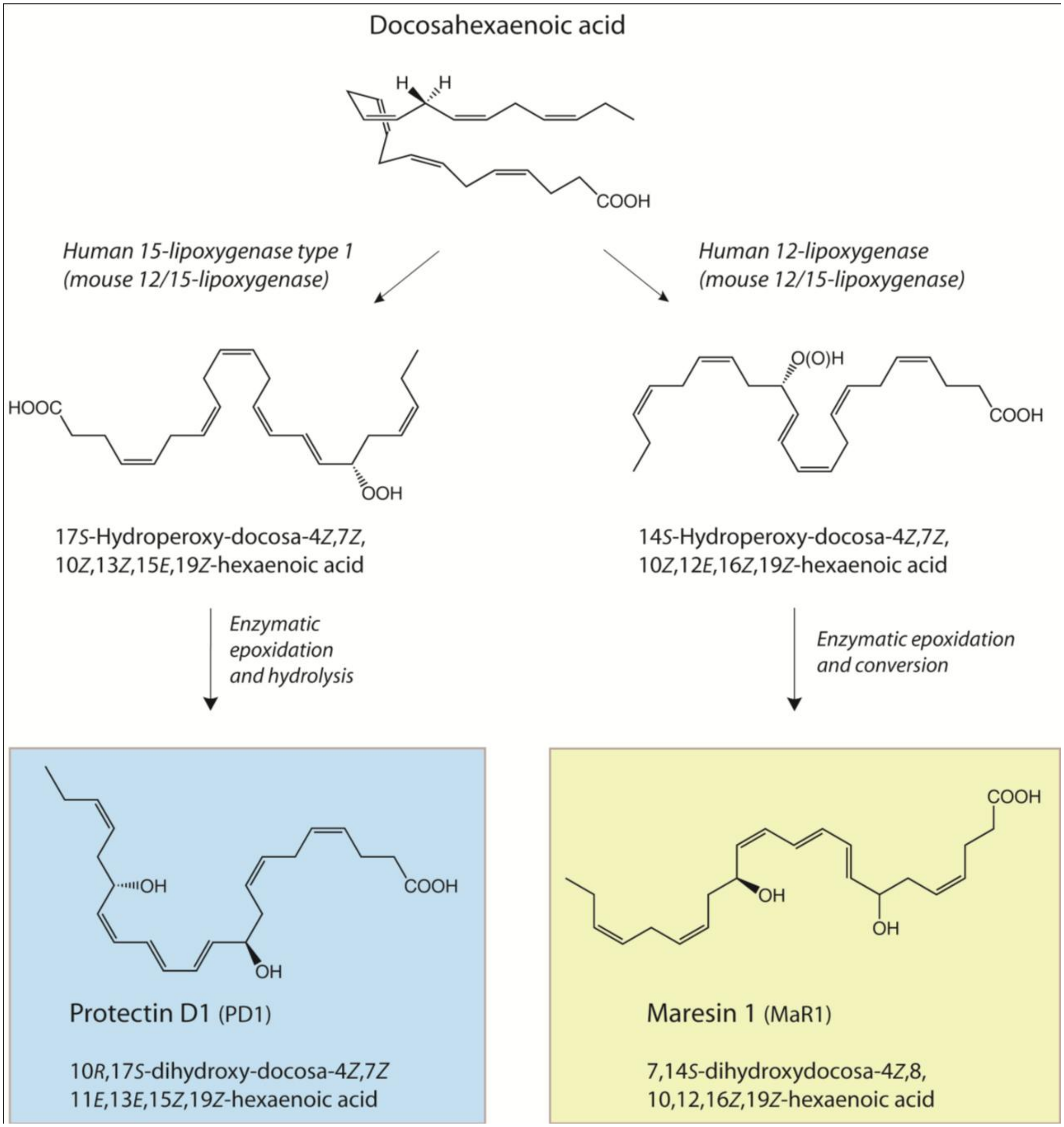

**FIGURE 5.** The formation of PD1 and MaR1. The lipoxygenation of DHA at the C17 or C14 position produces two families of anti-inflammatory and proresolving lipid mediators containing a triene moiety, which are therefore named docosatrienes. The hydroperoxide 17S-hydroperoxydocosa-(4*Z*,7*Z*,10*Z*,13*Z*,15*E*,19*Z*)-hexaenoic acid is used to form an intermediate epoxide that is hydrolyzed to the 10*R*,17*S*-dihydroxydocosatriene, PD1. In a similar manner, MaR1 (7,14*S*-dihydroxydocosa-(4*Z*,8,10,12,16*Z*,19*Z*)-hexaenoic acid) is formed from 14*S*-hydroperoxydocosa-(4*Z*,7*Z*,10*Z*,12*E*,16*Z*,19*Z*)-hexaenoic acid via an intermediate epoxide and rearrangement.

## *ω-3 PUFA–Derived Cyclopentenone-Containing Products*

Recently, ω-3 PUFA–derived lipid peroxidation products containing an electrophilic cyclopentenone moiety have also been shown to possess anti-inflammatory actions, akin to cyclopentenone-containing lipid oxygenation products derived from AA/$PGD_2$[215].

## Metabolic Inactivation of Lipid Mediators

Understanding the actions of endogenous lipid mediator agonists not only requires a biochemical description of their biosynthesis and actions, but also an appreciation of the pathways whereby such compounds are degraded. For a receptor agonist to act at physiologically relevant concentrations, local tissue levels need to reach concentrations close to the dissociation constant of their respective receptor, commonly in the low nanomolar range for G-protein coupled receptors. Several enzymatic pathways operate in lipid mediator degradation and play an important role in regulating their tissue levels, and may provide directional release from cells[216]. It has to be noted that marked species differences are known in the activities of the various metabolic routes and their relative importance in determining lipid mediator stability.

The dehydrogenation of secondary alcohol groups found in prostaglandins, lipoxins, and resolvins to the respective aldehyde by PGDH constitutes a major route of transformation of lipid mediators to, often, inactive metabolites. Both skin and lung are known to possess high activity, but the enzyme is known to play a regulatory role in many cells and tissues[217,218]. For example, dehydrogenation by PGDH reduces the 15-hydroxy group of $LXA_4$ to form inactive 15-oxo-$LXA_4$[219]. The same enzyme transforms $LXB_4$ predominantly to 5-oxo-$LXB_4$[78]. Dehydrogenation is often followed by reduction of a double bond to the respective dihydro compound by the enzyme 15-oxoprostaglandin 13-reductase ($LTB_4$ 12-hydroxydehydrogenase); in the case of 15-oxo-$LXA_4$, the C13-C14 double bond is reduced to form 13,14-dihydro-15-oxo-$LXA_4$[219]. Keto-prostaglandins and keto-dihydro-prostaglandins formed by these enzymes are effectively excreted in urine[220]. α,β-Unsaturated keto-prostaglandins can be enzymatically conjugated to glutathione as well, increasing water solubility and facilitating excretion[221].

Dehydrogenation of RvE1 by PGDH results in the formation of inactive 18-oxo-RvE1[191]. Dehydrogenation of the C12 alcohol enables reduction of the C10-C11 double bond to form inactive 10,11-dehydro-RvE1. 15-Hydroxyprostaglandin dehydrogenase transforms RvD1 into the biologically inactive 17-oxo-RvD1, which does not retain anti-inflammatory properties, and into 8-oxo-RvD1, which is as equally efficacious an anti-inflammatory compound as RvD1[202]. AT-RvD1 is a poor substrate for PGDH, as observed for other epimeric anti-inflammatory lipid mediators. Consequently, AT-RvD1 possesses approximately one log order greater potency in reducing total leukocyte infiltration in murine peritonitis when compared with RvD1[202]. This reinforces the concept that structural integration of resistance to degradation can enhance the anti-inflammatory activity of D-series resolvins, as observed for ATL.

Cytochrome P450–mediated ω-oxidation and ω-1 oxidation generally leads to inactivation of lipid mediators[222]. However, in the degradation of RvE1, the formed 20-hydroxy-RvE1 can maintain anti-inflammatory and proresolving activity[189]. The subsequent formation of 20-carboxy-RvE1 does lead to inactivation[189]. Incorporation of a fluorophenoxy group at the ω-end of lipoxins, RvE1, and RvD1 has been shown to reduce degradation and lowers dehydrogenation of the most distal hydroxy group. β-Oxidation is another pathway that operates in the degradation of lipid mediators via two-carbon-chain shortening from the α-end of lipid mediators[223]. Incorporation of a 3-oxa moiety that impedes β-oxidation has been shown to afford additional metabolic stability of lipoxins with retention of biological activity[224]. A large number of lipid mediator analogs have also been described in the patent literature; those of $LXA_4$ have recently been reviewed[225]. Medicinal chemistry has now achieved considerable success synthesizing stable lipid mediators that incorporate more than a few chemical features that afford metabolic stability towards several metabolic routes simultaneously[224,226].

## THERAPEUTIC APPLICABILITY OF ANTI-INFLAMMATORY LIPID MEDIATORS

A detailed examination of the inflammatory response has revealed that specific lipid mediators are formed at precise time intervals during the inflammatory response[108,111]. The paradigm that has emerged from these studies is that endogenously formed counter-regulatory lipid mediators, derived from AA, EPA, and DHA, actively regulate inflammation-resolution programs[111,186,227]. The magnitude and duration of the different phases of the inflammatory response have been defined in a set of indices that enable the determination and discrimination of anti-inflammatory and proresolving actions induced by specific lipid mediators[111]. The determination of resolution indices for $LXA_4$, RvE1, and PD1 has pointed to fairly independent regulation of anti-inflammation and resolution[27,111]. Our current understanding of the molecular mechanisms of the anti-inflammatory, proresolution, tissue-protecting, and host defense–activating functions of PUFA-derived lipid mediators permit an evaluation of the various approaches that have been taken, and may be further explored, towards achieving therapeutic applicability of anti-inflammatory/proresolution lipid mediators.

## Administration of PUFAs as Substrate for Endogenous Anti-Inflammatory/ Proresolution Lipid Mediator Biosynthesis

The endogenous biosynthesis of anti-inflammatory lipid mediators can be increased by providing additional exogenous EPA and DHA through increased dietary consumption or by systemic administration. Strategies include increased dietary intake via changes in diet or by ingestion of capsules containing such fatty acids, either as ethyl esters or acylated in phospholipids and/or triglycerides[179]. Parenteral administration is achieved by intravenous alimentation with ω-3 fatty acid–rich fish oil–derived or defined lipid emulsions[228,229]. Although a wealth of studies have been published that illustrate the salutatory effects of dietary ω-3 PUFA in animals and men, it is only recently that changes in intake have been related to changes in endogenous levels of specific ω-3 PUFA–derived resolvins and to their anti-inflammatory actions. One of the first suggestions that augmentation of exogenous ω-3 PUFA might be suitable for the treatment of inflammatory disease was to enhance endogenous PD1 formation for the treatment of Alzheimer’s disease[230]. The intravenous administration of albumin-bound DHA has been shown to afford protection against tissue damage and inflammation in brain I/R[231]. DHA administered by continuous subcutaneous infusion during kidney I/R allows the generation of detectable levels of D-series resolvins and PD1 in the kidney and plasma of mice[197]. Consumption of a high ω-3 fatty acid diet leads to an increased endogenous renal formation of PD1 in mice, and increasing dietary intake of DHA also prevented kidney I/R injury[198]. Furthermore, increased biosynthesis of E- and D-series resolvins and PD1 has been measured in rat bone marrow after dietary supplementation with DHA- and EPA-ethyl esters[232].

The direct evidence for the consideration that the salutatory roles of ω-3 PUFAs are mediated by ω-3 PUFA–derived anti-inflammatory mediators has been brought together by two principal observations. First, many of the anti-inflammatory actions that have been determined for resolvins and PD1 can be recapitulated by dietary provision of EPA and/or DHA. Second, augmentation of endogenous ω-3 fatty acid biosynthesis has been achieved in transgenic mice (*Fat-1* mice) that express a *Caenorhabditis elegans* ω-3 desaturase (the *fat-1* gene), and leads to markedly increased tissue levels of the ω-3 fatty acids, RvE1, RvD3, and PD1 in their colons[163]. Accordingly, these mice display a pronounced protection from experimentally induced colon inflammation and tissue damage, as well as a markedly reduced acute lung injury[163,233].

Recent studies have further examined the protective actions of dietary ω-3 fatty acids in relationship to modifying lipid mediator formation. In a model of chemical carcinogenesis, a low ratio of ω-6/ω-3 fatty acid consumption was shown to protect against tumor growth in lungs of mice. Of interest, an association between 15-lipoxygenase expression and the extent of tissue protection by fish oil consumption was observed[234]. Furthermore, enhanced provision of dietary ω-3 fatty acids has been shown to reduce the biosynthesis of proinflammatory eicosanoids; for example, the formation of $LTB_4$ in liver is decreased by increased dietary DHA and reduces carbon tetrachloride–induced necroinflammatory liver injury in mice[235].

Bone marrow–formed resolvins and protectins have been suggested to constitute a source of anti-inflammatory and proresolving mediators that may regulate stem cell differentiation[236,237], but may also be mobilized to the peripheral circulation. Specific signals are believed to exist that activate the mobilization and delivery of ω-3 fatty acids from the liver to the brain and retina when required[238]. The rapid appearance of free ω-3 EPA and DHA at sites of inflammation has been demonstrated to occur concurrently with the development of edema and leukocyte infiltration[227], which indicates that the required substrate for resolvin and protectin biosynthesis can be provided via the circulation at the early stages of an inflammatory response. Dietary supplementation with ω-3 fatty acids may play a role in replenishing stores of these fatty acids when demand increases; for example, during an inflammatory response in a particular organ. A recent study has indicated that even hospitalized patients consuming a diet rich in ω-3 PUFAs are significantly less likely to suffer from the inflammatory sequelae of acute lung injury/ARDS[239]. Large decreases in levels of EPA in serum triglycerides are furthermore observed during allergic sensitization in mice that can be largely prevented by dietary DHA supplementation, suggesting that the circulation can rapidly and sensitively accommodate changes in ω-3 PUFA levels in response to changes in tissue homeostasis[240].

Whereas dietary supplementation with the ω-3 PUFAs EPA and DHA can clearly afford benefits for human health, the provision of increased intake of exogenous AA to specifically enhance the formation of AA-derived anti-inflammatory lipid mediators has not been seriously considered, probably given the already lavish intake of AA via our diet. Recent studies employing dietary augmentation of ω-3 PUFAs have indicated, however, that AA-derived anti-inflammatory lipid mediators may also constitute a significant mechanism of anti-inflammatory tissue protection with which excess ω-3 PUFA intake can interfere. For example, increasing the dietary supplementation with ω-3 PUFAs has been shown to cause a reduction in the capacity of the airways to form protective $PGE_2$ during ovalbumin-sensitive asthma in mice, and lead to increased proinflammatory cytokine release[241]. Recent studies have also pointed out that ω-6 PUFAs other than AA, as well as additional ω-3 PUFAs, need to be taken into consideration as potential substrates for the formation of additional anti-inflammatory lipid mediators. Both ω-6 docosapentaenoic acid (DPA) and ω-3 DPA have been demonstrated to be good substrates for 5-, 12-, and 15-lipoxygenases to form mono- and dihydroxylated products[242,243]. Two of the ω-6 DPA–derived products, ω-6 17*S*-hydroxypentaenoic acid and ω-6 10,17-dihydroxy-(4*Z*,7*Z*,11*E*,13*Z*,15*E*)-pentaenoic acid, formed by the single and double oxygenation of ω-6 DPA by soybean 15-lipoxygenase, respectively, were shown to be potent anti-inflammatory lipid mediators[243]. Omega-6 17*S*-hydroxypentaenoic acid was shown to be present in blood, cardiac tissue, and trachea in rat, and can be formed in human blood from ω-6 DPA[242].

## Mechanism-Based Therapy using Anti-Inflammatory/Proresolution Mediators and Their Stable Analogs

The oral or systemic administration of ω-3 PUFAs may not be generally applicable to the treatment of inflammatory disease. In specific tissues, the general supplementation with ω-3 PUFAs may potentially interfere with the formation of endogenous AA-derived counter-regulatory lipid mediators[241] Direct pharmacological intervention with specific members of the previously described anti-inflammatory/proresolving lipid mediators, or their stable analogs, could provide an appealing targeted or mechanism-based approach for the treatment of inflammation. To appreciate the use of these compounds as drugs, a good understanding of their mechanisms of action is required and, ideally, the relationship between pharmacokinetics and *in vivo* activity needs to be defined. The following requirements call for consideration for therapeutic applicability of lipid mediators and their analogs.

First of all, chemical and physical stability is required for prior processes such as chemical synthesis, formulation, and storage. Many lipid mediators are sensitive to light-, heat-, and acid-stimulated double bond isomerization, as well as to oxidation, with small changes in chemical structure leading to complete loss of bioactivity. Reduced exposure to oxygen, heat, and incident light is generally required to avoid degradation during formulation and storage. The incorporation of specific functional groups that prevent chemical degradation may facilitate pharmaceutical development[155,244,245,246]. The introduction of structural modifications that lock cis-double bonds in a rigid configuration can diminish the loss of activity due to isomerization. This has been achieved by the introduction of an acetylenic bond between carbons 11 and 12 in the 15-epi-$LXA_4$ structure; for example, in the trienyne-containing analog of ATLa2 ZK-994 (compound **2**, Fig. 3)[247,248]. The introduction of a benzo structure fused to the carbon 11-12 cis double bond in $LXA_4$ has recently also been shown to maintain anti-inflammatory and proresolution activity (compound **3**, Fig. 3)[246,249].

Second, the applicability of PUFA-derived lipid mediators as anti-inflammatory/proresolution drugs is constrained by enzymatic degradation, which can occur after administration. Hence, the structural modification of anti-inflammatory lipid mediators to confer enhanced metabolic resistance can afford new and more attractive pharmacokinetic properties, such as reduced clearance, increased concentration in blood, and increased pharmacological efficacy[224,250]. In addition to enzymatic degradation occurring at local tissue level, the systemic administration of lipid mediators often leads to rapid uptake and subsequent enzymatic inactivation during passage through the pulmonary circulation[251], a major determinant to therapeutic applicability of lipid mediators. In particular, the activity of PGDH readily dehydrogenates secondary alcohol groups to the corresponding aldehydes, e.g., present at C15 (prostaglandins and $LXA_4$), C17 (17*S*-resolvins), or C18 (E-series resolvins). Resistance to inactivation by dehydrogenation is afforded by the presence of an epimeric alcohol group, e.g., in 15-*epi*-$LXA_4$ and the aspirin-triggered E- and D-series resolvins (Fig. 1). Increased metabolic stability has also been achieved by introduction of substituents proximal to the alcohol-bearing carbon, interfering with the dehydrogenase-catalyzed oxidation. For example, resistance to PGDH has been achieved by incorporation of one or two methyl groups at the alcohol-bearing carbon, e.g., in 15-methyl-$PGE_1$[252] or 15-methyl-$LXA_4$[253]). $PGE_2$ analogs with methyl substitutions at carbon 15 or 16, which resist PGDH-mediated degradation, can potently activate anti-inflammatory actions in the large intestine[254]. Misoprostol, a stable analog of $PGE_2$, has a hydroxyl group (and additional methyl group) placed at C16 instead of C15, resists PGDH-mediated enzymatic degradation, and displays tissue-protective actions in the stomach and anti-inflammatory activity on neutrophils (compound **4,** Fig. 3)[255]. A dehydrogenation-resistant RvD1 analog, 17-(*R*/*S*)-methyl-RvD1 carboxy methyl ester (compound **5,** Fig. 3), is able to activate a potent anti-inflammatory action in second-organ lung injury after hind limb I/R (~5 μg/kg i.v.), even when administered at a significantly long period after reperfusion[227].

A second important route of enzymatic degradation is cytochrome $P_{450}$–mediated oxidation of the ω-carbon or ω-1 carbon atom[223]. Abolition of oxidation can be accomplished by introduction of a distal substituent. The introduction of distal substituents, such as a *para*-fluoro-phenoxyl or a phenyl group, has been used successfully to abolish oxidation. Retention of pharmacological activity has been demonstrated, e.g., with 16-(*p*-fluorophenoxy)-15-epi-$LXA_4$-methyl ester (ATLa2; compound **1,** Fig. 3)[253] and 19-(*p*-fluorophenoxy)-RvE1-methyl ester (compound **6,** Fig. 3)[191]). In specific cases, enhanced efficacy *in vivo* has been achieved. For example, 19-(*p*-fluorophenoxy)-RvE1 carboxy methyl ester reduces acute lung inflammation in a murine model of I/R second-organ injury (hind limb I/R), whereas the same dose (~50 μg/kg i.v.) of native RvE1 does not demonstrate appreciable actions in this model[227]. It is thought that modification of the ω-end of the lipid mediator structure not only precludes ω-oxidation, but may also reduce the rate of inactivation by PGDH.

Nevertheless, even inhibition of PGDH-mediated dehydrogenation and avoidance of ω-oxidation does not turn the 15-epi-$LXA_4$ stable analog ATLa2 into a completely stable analog, and still displays a $t_{½}$ of <20 min in blood after i.v. administration in the mouse[155,253]. This indicates that additional metabolic pathways can operate *in vivo*. The activity of the β-oxidation system is likely to be of importance[155]. Inhibition of this route of degradation has been achieved by replacement of carbon 3 with an oxygen atom. 3-Oxa-analogs of 15-*epi*-$LXA_4$, which resist β-oxidation, demonstrate anti-inflammatory activity and display a $t_{½}$ >1 h (e.g., the 3-oxa-trienyne analog of ATLa2; compound **2,** Fig. 3)[155]. Of interest, the recently evaluated ω-6 DPA–derived products 17*S*-hydroxy-DPA and ω-6 10,17-dihydroxy-DPA appear to be comparatively stable after systemic administration in the rat[242]. This finding suggests that both chain length and position of the terminal double bond are also important determinants in the recognition of AA-, DHA-, and DPA-derived lipid mediators by degradative pathways.

Adequate absorption and distribution after administration is also important for pharmaceutical applicability. A favored route of administration of lipid mediator drugs is topical application for the treatment of superficial organs, such as skin and eye, e.g., as ointments and eye drops, thereby avoiding systemic metabolic degradation. The topical administration of an isopropyl ester RvE1 analog RX-10045 (Resolvyx Pharmaceuticals) has recently been shown to reduce choroidal neovascularization successfully after laser-induced injury of the retina[209,210,211], indicating that sufficient absorption and distribution in the eye occurred. A phase I/II clinical trial to assess the potential protective actions of RX-10045 in the ocular surface of human patients with dry eye has recently been completed (Clinicaltrials.gov identifier: NCT00799552). The topical applicability of isopropyl ester analogs of lipid mediators in the eye has been carefully determined for the $PGF_2\alpha$-analog latanoprost (compound ***7***, Fig. 3), which is used for the treatment of glaucoma[256]. The topical administration of this isopropyl ester on the cornea allows distribution even into the anterior portion of the eye, with the cornea serving a depot function from where the active compound is slowly released[256,257]. The suitable pharmacokinetic properties in the eye of lipid mediator isopropyl esters are thus translatable to obtaining therapeutic intraocular applicability, e.g., for novel resolvin isopropyl esters. The anti-inflammatory actions of ATLa2, $PGE_{1,}$ and analogs of $PGE_1$ after topical application on the skin have also been demonstrated in the treatment of several types of skin inflammation[258,259].

The oral route of administration is also a suitable approach for administration of anti-inflammatory lipid mediators. Oral administration of the $PGE_1$ analog 15*S*-methyl-$PGE_1$ can stimulate systemic anti-inflammatory actions, as demonstrated by protection from immune complex–induced vascular injury and adjuvant-induced arthritis in rats[252]. Lipoxin analogs are orally available for systemic anti-inflammatory actions and their pharmacokinetic properties can be improved by incorporation of metabolic resistance[155,248]. ATLa2 also displays pronounced anti-inflammatory and tissue-protective actions in the gastrointestinal tract mucosa after oral intake[260]. It is expected that bioavailability after oral administration is constrained by the success to avoid first-pass metabolism by ω-oxidation and β-oxidation activities in the liver. However, few studies have specifically addressed the fate of orally administered lipid mediators or their analogs, and their precise route of absorption from the gastrointestinal tract is largely undescribed.

In addition to enhanced metabolic stability, a change in distribution due to altered transport of chemically modified lipid mediators may be employed for changing the pharmacokinetic properties of specific lipid mediators. The lung has been shown to function as a depot tissue for the stable $PGE_2$ analogs (15S)-15-methyl $PGE_2$ methyl ester and 16,16-dimethyl $PGE_2$; the compounds are actively transported from the pulmonary circulation into lung tissue and are slowly released back to the circulation without undergoing metabolic inactivation by PGDH[261]. The targeted delivery of a prostaglandin to induce tissue-specific protective actions has been achieved with $PGE_1$ in the form of a polymeric prodrug[262]. The authors achieved sufficient metabolic stability by formation of a hydrazone bond of the cyclopentane carbonyl group of $PGE_1$ with a galactosylated polyglutamine hydrazine derivative, which is targeted to the liver after uptake by the galactose receptor. The targeted accumulation of the $PGE_1$ conjugate in liver parenchyma stimulated a hepatoprotective action against carbon tetrachloride–induced hepatotoxicity, whereas parenteral administration of free $PGE_1$ did not show any protection.

It needs to be pointed out that in some cases, the native, chemically unmodified lipid mediator has been shown to be efficacious. For example, $LXA_4$ applied topically can still reduce inflammation in the anterior part of the eye[263]. $LXA_4$ is equally as efficacious after intragastric dosing on zymosan-stimulated peritonitis in mice as some metabolically stable analogs[248]. Such results indicate that general conclusions on pharmacokinetic-pharmacodynamic relationships for lipid mediator–based drugs are currently difficult to formulate, and need to be established experimentally for each application in consideration with additional needs for structural changes to address chemical stability. In summary, a range of studies have now indicated that it is possible to employ lipid mediators for the treatment of inflammation and that multiple routes of administration are available to achieve potential therapeutic benefit.

With respect to understanding the mechanisms of action of counter-regulatory lipid mediators in inflammation, it is of interest to note that both $LXA_4$ and RvE1 have been shown to act as agonists on one receptor to activate distinct anti-inflammatory/proresolution actions, and also act as a receptor antagonist or partial agonist on a different receptor to counteract the binding and action of a specific proinflammatory mediator. $LXA_4$ inhibits the binding of $LTD_4$ to the CysLT1 receptor[264], thereby reducing the proinflammatory actions of cysteinyl leukotrienes responsible for vascular permeability changes (e.g., in peritonitis) as well as eosinophilic inflammation[265,266]. RvE1 inhibits the binding of $LTB_4$ to the BLT1 receptor, thereby diminishing the proinflammatory actions of $LTB_4$. BLT1 receptor activation by $LTB_4$ plays a significant role in maintaining a proinflammatory environment, and smooth muscle and endothelial cell activation, in atherosclerosis, myocardial infarction, rheumatoid arthritis, bronchial asthma, and I/R injury[266,267,268,269,270]. Treatment with (analogs of) $LXA_4$ or RvE1 may therefore be expected to promote resolution of inflammatory disease characterized by strong cysteinyl leukotriene or $LTB_4$ components, respectively. As specific binding sites for other anti-inflammatory lipid mediators will become identified, it will be of interest to determine if this twofold receptor action is a fundamental mode through which proresolution lipid mediators function to direct the inflammatory response towards resolution. It also suggests that in order for novel drugs to activate resolution effectively, success may depend on adherence to this principle of activating anti-inflammatory/proresolution pathways, while concurrently dampening a dominating proinflammatory pathway. In addition, $LXA_4$ has been shown to reduce $LTB_4$ formation, indicating that $LXA_4$ down-regulates the $LTB_4$ pathway at multiple levels[271].

Another potential pharmacological approach to the initiation of an anti-inflammatory/proresolution response is the activation of receptors for ω-3 fatty acid–derived lipid mediators by agonists that are not based on the chemical structure of the lipid mediator ligand. Oligopeptide ligands, derived from the chemoattractant protein chemerin, activated CMKLR1, and can potently reduce macrophage activation and stimulate the reduction of murine inflammation with high potency and efficacy[272]. Such ligands are also amenable to modification, enabling the development of metabolically stable peptides that maintain their anti-inflammatory activity[272,273]. A number of nonlipid/nonpeptide ligands for the $LXA_4$ receptor FPR2/ALX have also been developed, some of which activate anti-inflammatory actions observed with $LXA_4$[225,274].

The employment of reactive lipid mediators based on the chemical structure of $PGD_2$-derived cyclopentenones is being actively evaluated for potential therapeutic purposes. It is challenging to envisage the pharmaceutical applicability of those anti-inflammatory lipid mediators that are inherently chemically reactive, such as the $PGD_2$ dehydration products and PUFA-derived lipid peroxidation products containing an α,β-unsaturated aldehyde. Such enones have been shown to exert anti-inflammatory activity not only locally[275], but even at sites remote from the site of administration. For example, i.p. administration of 15-deoxy-$\Delta^{12,14}$-$PGJ_2$ can reduce inflammation of bladder, pleura, or joints in experimental animals[118,276]. A certain degree of specificity in electrophilic reactivity of cyclic enones with various regulatory proteins exists, and is defined by the structural environment and nucleophilic strength of the reacting sulfhydryl groups in a given protein, as well as by levels of competing nucleophiles such as glutathione[277,278]. It has been demonstrated that addition reactions of cyclic enones to thiols can be reversible[279]. In consequence, it is conceivable that reaction with high-abundance sulfhydryl-containing compounds, such as GSH and albumin, mediates transport and delivery of the respective thioether adducts to specific domains of regulatory proteins where subsequent release of the enone is favored, followed by reaction with a reactive sulfhydryl residue, leading to a change in protein function.

Recent advances employing targeted delivery of cyclopentenone lipid mediators do appear to have overcome the technical challenge to provide control over administration and delivery to a specific tissue; a marked reduction in experimental atherosclerosis in mice has been achieved using liposome-protected cyclopentenone-containing lipid mediators targeted to inflamed vasculature[280]. A number of synthetic cyclopentenone analogs have also been developed that retain anti-inflammatory action[275,281]. In addition, a number of natural and synthetic cyclopentenone-containing lipid mediators have been identified to possess potent biological activity and constitute an interesting pool of potential anti-inflammatory molecules[282,283,284].

## Modulation of Endogenous Pathways for Counter-Regulatory and Proresolution Lipid Mediator Biosynthesis

Recent research has indicated that the aspirin-driven change in stereospecificity of PUFA oxygenation may constitute a pharmacological equivalent of the endogenous manner in which COX-2 can redirect the oxygenation of AA from forming $PGH_2$ towards 15(*R*)-HETE. NO, possibly by S-nitrosylation of a critical cysteine residue in COX-2, can trigger the formation of epimeric oxygenation products such as 15-epi-$LXA_4$[159,160]. It is likely that the endogenous formation of aspirin-triggered E- and D-series resolvins (e.g., 18(*R*)-HEPE from EPA, and 17(*R*)-HDHA from DHA) can also be initiated by this NO-mediated switch. The concomitant expression and/or activation of COX-2 and a NO synthase during inflammation may constitute a simple, yet powerful, way in which the body can swiftly increase the metabolic stability and endogenous efficacy of anti-inflammatory/proresolution mediators to control the extent of inflammation or promote the progress of the response to resolution. In this respect, it is important to restate the role of COX-2 as a key enzyme in the initiation of inflammation resolution[113,285]. Given the recognized number of anti-inflammatory/proresolution lipid mediators that can be formed by COX-2, pharmacological inhibition of its activity is likely to interfere with endogenous control of inflammation and is expected to compromise the capacity for resolution[286,287]. If this enzyme needs to be modulated pharmacologically, it appears more rational to stimulate the “epi-oxygenase” activity of this enzyme transiently during inflammation, e.g., employing NO-donating drugs, although this approach has not been generally evaluated for its usefulness *per se*.

In addition to enabling the formation of 18*R*-HEPE, the aspirin-triggered conversion of EPA also permits the formation of 15*R*-HEPE, which is transformed by 5-lipoxygenase to 15-epi-$LXA_5$, a structural analog of 15-epi-$LXA_4$[184]. Other common anti-inflammatory drugs, such as acetaminophen and indomethacin, can also permit COX-2 to form small quantities of 18*R*-HEPE[184], indicating that traditional anti-inflammatory drugs may play hitherto unappreciated roles in the biosynthesis and actions of proresolving $\omega$-3 fatty acid–derived lipid mediators. In this respect, a highly interesting development has started that comprises a re-evaluation of many successful, both old and new, drugs for potential proresolution properties. Ajulemic acid, a synthetic anti-inflammatory and analgesic cannabinoid with low toxicity, stimulates endogenous $LXA_4$ and 15-deoxy-$\Delta^{12,14}$-$PGJ_2$ generation, and promotes resolution[288,289]. Oxymetazoline, a well-known nasal decongestant, stimulates $LXA_4$ biosynthesis and down-regulates $LTB_4$ formation and respiratory burst in human neutrophils[290]. Losartan, an angiotensin II receptor antagonist, has been found to activate a $PGE_2$-mediated anti-inflammatory action in the lung and reduces bleomycin-induced pulmonary fibrosis[291]. Such studies indicate that modulation of endogenous counter-regulatory lipid mediator biosynthesis has already been achieved by existing pharmacotherapy (be it unknowingly when these compounds were developed). Appreciation of the contributions of proresolution activities activated by known drugs is likely to provide us with additional insights into the ways counter-regulatory lipid mediator biosynthesis is regulated *in vivo*.

## Modulation of Anti-Inflammatory/Proresolution Lipid Mediator Action

Another way in which lipid mediator action can be modified is via modulation by other counter-regulatory mediator pathways. In the cornea, the heme oxygenase-carbon monoxide (CO) anti-inflammatory system acts in close cooperation with $LXA_4$ to maintain a rapidly healing tissue environment[292]. In a murine model of acute lung injury, the anti-inflammatory actions of ATLa2 are mediated in part via activation of CO biosynthesis[293]. 15-Epi-$LXA_4$ can act in concert with another endogenous anti-inflammatory mediator, inosine monophosphate, to regulate neutrophilic inflammation[294]. Some of the actions of $LXA_4$, 15-epi-$LXA_4$, and RvD2 have been shown to be mediated by activation of NO synthesis[62,205,295]. The nitration of unsaturated fatty acids by NO-dependent free-radical reactions constitutes a means to forming nitro-alkenes, which have been shown to exert signaling functions through release of NO, as well as by alkylation of regulatory sulfhydryl groups in specific enzymes and regulatory proteins. Of relevance, some nitro-fatty acids also exert anti-inflammatory activity[296,297]. Annexin 1, a peptide derived from the glucocorticoid-stimulated anti-inflammatory protein lipocortin, also activates FPR2/ALX, indicating that part of the anti-inflammatory actions of glucocorticoids converge at this point with anti-inflammatory lipoxin signaling[298]. Glucocorticoids can also activate protection of cardiac tissue after I/R injury through stimulation of $PGD_2$ biosynthesis[299]. Another recent example has been provided by a study that indicated that the neurosteroid 7β-hydroxy-epiandrosterone potently ameliorates experimental colitis in rats via activation of COX-2 expression and shifting prostaglandin formation from $PGE_2$ to $PGD_2$ and the cyclopentenone 15-deoxy-$\Delta^{12,14}$-$PGJ_2$[300]. In summary, a number of studies point to a close interplay between PUFA-derived anti-inflammatory/proresolving lipid mediators and additional recognized anti-inflammatory mechanisms. It remains to be fully assessed how to take advantage of such interactions for therapeutic benefit. Of interest, a recent study has indicated that even natural compounds with known anti-inflammatory properties and that are safe for human consumption can synergistically enhance the anti-inflammatory actions of the ω-3 PUFAs EPA and DHA[301].

## Challenges and Opportunities

In order to be able to treat inflammatory disease with anti-inflammatory/proresolution mediators, we need to learn more about the specific differences and tissue-specific functions of the various described lipid mediators. A number of potential molecular defects with respect to lipid mediators may contribute to inflammatory disease: (1) an altered endogenous capacity to form adequate amounts of specific counter-regulatory lipid mediators, (2) defects in the molecular components for correct signal transduction to particular lipid mediators, or (3) the mounting of an inflammatory response that is not suitable for clearing the inciting stimulus. We currently possess modest information on the specific molecular defects that may underlie inflammatory disorders. Hence, it is still a largely open issue to estimate and determine which specific lipid mediator/analogs would be most applicable for possible therapeutic use and at which specific time-point during the inflammatory response their employment would be suitable.

In a recent study, it was pointed out that a large reduction in FPR2/ALX expression is observed in patients with asthma, and which contributes to impaired $LXA_4$ signaling[302]. Defects in counter-regulatory signaling by anti-inflammatory lipids have also been reported in scleroderma lung disease and are actively being evaluated in cystic fibrosis[303,304,305]. In neutrophils from individuals with the congenital disease “familial localized aggressive periodontitis”, RvE1 can potently down-regulate neutrophil activation, but counter-regulatory signaling in response to $LXA_4$ is defectuous[306]. Furthermore, a defect in the normal execution of resolution also appears to play an important role in atherosclerotic lesions[204]. In Alzheimer’s disease, the capacity for PD1 biosynthesis is decreased[230]. Neutrophils from chronic granulomatous disease patients undergo a reduced rate of apoptosis and when eliminated by macrophages from these patients, phagocytosis is not accompanied by release of anti-inflammatory mediators such as $PGD_2$ and TGF-β[307].

Taken together, these observations indicate that distinct defects in counter-regulatory lipid mediator formation and signaling are present in different chronic inflammatory settings[54]. The studies reported to date indicate that future development of resolution therapeutics will require the identification of the functional circuits for resolution that can still be activated in specific inflammatory disorders. The choice of a particular anti-inflammatory lipid mediator and timing of administration may present a further challenge to applicability of mechanism-based lipid mediator therapeutics. As an example, cysteinyl leukotrienes can activate the CysLT1 receptor promoting the development of increased vascular permeability during the proinflammatory phase of inflammation. Antagonism of CysLT1 could thus be considered a therapeutic target to reduce the development of inflammatory edema[265,308]. However, it has been shown that in experimental chronic pulmonary inflammation induced by bleomycin, the CysLT1 receptor is important in the counter-regulation of inflammation and inhibition of its function promotes inflammation leading to fibrosis[309,310]. The use of stable analogs of $LXA_4$ to stimulate FPR2/ALX and antagonize CysLT1 simultaneously would be helpful to drive resolution in an acute inflammatory setting, but would be potentially harmful in certain types of chronic inflammation.

Interestingly, some of the known compounds that activate anti-inflammatory lipid mediator formation, such as EPA/DHA, stable lipoxin analogs, and ajulemic acid, have a very favorable safety profile at doses considered suitable for treatment. Activation of endogenous anti-inflammation/resolution is, in fact, a way in which the body actively promotes tissue protection. Does this mean that drugs with resolution-promoting activity are inherently harmless? This will be an important question to answer in future research. Aspirin itself sets off COX-2–mediated epi-lipoxygenation to form aspirin-triggered lipoxins and aspirin-triggered resolvins, but also interferes with both COX-1- and COX-2–mediated formation of eicosanoids that are necessary for correct function of the stomach[311]. It is possible that future development for new anti-inflammatory therapeutic approaches should aim for anti-inflammatory/proresolution drugs that do not inhibit cyclo-oxygenases at all, in order to preserve the capacity to generate endogenous lipid mediators that counter-regulate inflammation and turn it off. Therapeutic strategies for the treatment of inflammation should avoid any inhibition of key enzymes like COX-2 located at the top of a metabolic cascade and controlling many physiological functions. In this context, if the development of novel COX-directed strategies would be contemplated, the usefulness of reversible activation of COX-2 epimeric oxygenation to drive the formation of ATL and AT-resolvins would merit consideration.

In acute inflammation as a result of infection, it is conceivable that intervention with resolution-promoting compounds might lead to incomplete phagocytotic removal of microbes. We do not know yet whether pharmacological acceleration of resolution may bring along unanticipated problems such as defects in microbial clearance or inadequate tissue healing. Recent studies with RvD2 have indicated that promotion of resolution (in experimental sepsis) is accompanied by an increased rate of bacterial killing and phagocytosis[205]. This indicates that the rate of phagocytosis during a normal inflammatory response is not operating at maximum rate, and can be enhanced by increased monocyte recruitment and increased phagocytotic capacity. Augmentation of monocyte recruitment is precisely one of the key actions activated by anti-inflammatory/proresolution mediators[78]. Such enhanced capacity for clearance of infectious agents fits within a broader paradigm, namely the activation of host defense by specific lipid mediators. Also ATL has been shown to stimulate host defense by activating the expression of antibacterial defense such as bactericidal permeability–inducing protein in epithelial cells[312].

Finally, the applicability of anti-inflammatory/proresolution lipid mediators may be found in the treatment of inflammatory disorders known to be resistant to treatment with currently used anti-inflammatory drugs or by displaying fewer side effects[313]. For example, there is a need for drugs that can be used for long-term use as topical anti-inflammatory drugs instead of, for example, glucocorticoids. In addition, there may be specific cases where the development of novel anti-inflammatory/proresolution approaches is desirable, e.g., for the treatment of inflammatory disease characterized by hypersensitivity to specific anti-inflammatory drugs, such as aspirin[314]. Possibly, we may soon witness therapeutic resolution of some thus far intractable chronic inflammatory disorders.

## CONCLUSION

Knowledge of the regulatory actions of endogenous anti-inflammatory/proresolution PUFA-derived lipid mediators in the inflammatory response is currently expanding rapidly. The presented evaluation of existing and potential strategies to employ ω-3 and ω-6 PUFA-derived lipid mediators in the treatment of inflammatory disease indicates that active modulation of the inflammatory response is a promising avenue for innovative pharmacotherapy. Such approaches remain to be translated to humans. Specific aspects, such as the incorporation of resistance to metabolic degradation, strategies to target multiple receptors, a better understanding of molecular defects in lipid mediator formation and signaling in inflammatory disease, and a further appreciation of the proresolution actions of known anti-inflammatory and tissue-protective drugs, will further expand our understanding to rationally employ lipid mediator therapeutics for the treatment of inflammatory disease.
